# Bonding wood with uncondensed lignins as adhesives

**DOI:** 10.1038/s41586-023-06507-5

**Published:** 2023-08-08

**Authors:** Guangxu Yang, Zhenggang Gong, Xiaolin Luo, Lihui Chen, Li Shuai

**Affiliations:** College of Materials Engineering, Fujian Agriculture and Forestry University, Fuzhou, China

**Keywords:** Sustainability, Mechanical properties

## Abstract

Plywood is widely used in construction, such as for flooring and interior walls, as well as in the manufacture of household items such as furniture and cabinets. Such items are made of wood veneers that are bonded together with adhesives such as urea–formaldehyde and phenol–formaldehyde resins^1,2^. Researchers in academia and industry have long aimed to synthesize lignin–phenol–formaldehyde resin adhesives using biomass-derived lignin, a phenolic polymer that can be used to substitute the petroleum-derived phenol^3–6^. However, lignin–phenol–formaldehyde resin adhesives are less attractive to plywood manufacturers than urea–formaldehyde and phenol–formaldehyde resins owing to their appearance and cost. Here we report a simple and practical strategy for preparing lignin-based wood adhesives from lignocellulosic biomass. Our strategy involves separation of uncondensed or slightly condensed lignins from biomass followed by direct application of a suspension of the lignin and water as an adhesive on wood veneers. Plywood products with superior performances could be prepared with such lignin adhesives at a wide range of hot-pressing temperatures, enabling the use of these adhesives as promising alternatives to traditional wood adhesives in different market segments. Mechanistic studies indicate that the adhesion mechanism of such lignin adhesives may involve softening of lignin by water, filling of vessels with softened lignin and crosslinking of lignins in adhesives with those in the cell wall.

## Abstract (web summary)

A straightforward strategy for preparing lignin-based wood adhesives from lignocellulosic biomass is described, with the resulting adhesives demonstrating performance attractive for plywood manufacture.

## Main

Lignin–phenol–formaldehyde resin adhesives are generally synthesized by reacting a mixture of isolated lignin (for example, kraft, soda or biorefinery lignin) and phenol with formaldehyde under alkaline conditions^7^. Available commercial lignins have high molecular weights and a paucity of vacant reactive sites owing to substantial condensation during their extraction from biomass^8,9^. Lignin–phenol–formaldehyde resin adhesives have higher viscosity, are more deeply coloured and require more severe curing conditions than urea–formaldehyde and phenol–formaldehyde resin adhesives^10^, making them unattractive to plywood manufacturers. Additional physical and chemical treatments of lignins could ameliorate these issues but increase the production cost of lignin–phenol–formaldehyde resin adhesives^11^. Lignin–phenol–formaldehyde resin adhesives cannot compete in production cost with urea–formaldehyde resins, which dominate the wood adhesive market, and in performance with phenol–formaldehyde resins for preparing structural wood panels. The use of lignin to produce industrially applicable wood adhesives therefore remains challenging in biorefining and plywood manufacturing industries. Addressing this issue would promote not only the use of green adhesives but also the development of profitable biorefining schemes.

**Fig. 1:**
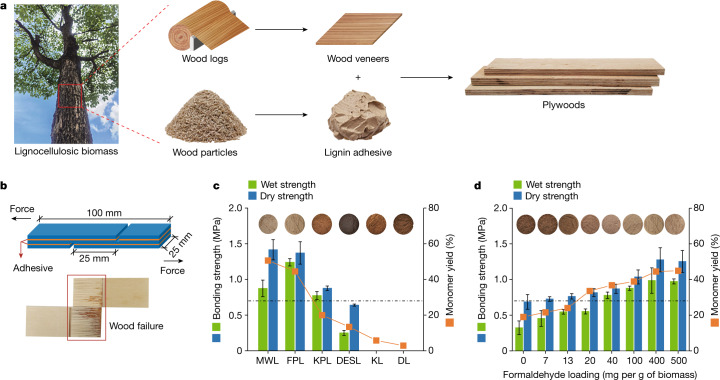
Adhesion performance of adhesives prepared from different lignins. **a**, Schematic illustration of preparation of plywood from wood veneers with lignins as adhesives. **b**, Schematic illustration of three-layer FPL-bonded plywood specimens used for adhesion performance tests (top) and wood failure of the specimen after a wet strength test (bottom). **c**, Adhesion performance of adhesives prepared from lignins isolated with different methods (KPL, acetone-protected lignin; DESL, deep eutectic solvent-extracted lignin; KL, kraft lignin; DL, dioxane–HCl lignin). Lignin adhesive preparation: 1:2 (w/w) lignin/water, pH 7; hot-pressing conditions: 190 °C, 8 min, 1.5 MPa and a glue application level of 100 g m^−2^. **d**, Effects of condensation degrees of FPLs on their adhesion performances. Lignin adhesive preparation: 1:2 (w/w) lignin/water, pH 7; hot-pressing conditions: 170 °C, 8 min, 1.5 MPa and a glue application level of 100 g m^−2^. The dot-dashed line at 0.7 MPa in **c**,**d** marks the minimum industrial requirement for the adhesion strength. All sample preparation and testing procedures as well as data calculation are described in the Methods. All examined bonding strengths were found to be significantly (analysis of variance (ANOVA), *P* < 0.01) affected by the condensation degree (or hydrogenolysis monomer yields) of lignins (Extended Data Table 1). Error bars show the s.d. of measured bonding strengths with four repeats.

In lignocellulosic biomass, lignin acts as a glue that provides strength to cell walls by effectively binding cellulose and hemicelluloses together^12^. When isolated at an elevated temperature and/or with an acidic or alkaline catalyst, lignin can condense to form interunit C–C linkages between its units^13,14^. We contend that condensation of lignin under an elevated temperature is similar to the curing of wood adhesives such as phenol–formaldehyde and urea–formaldehyde resins during hot pressing. Inspired by the similarity, we reasoned that lignin with no or limited condensation could be directly used as a wood adhesive, which may undergo crosslinking following hot pressing (Fig. 1a).

**Extended Data Fig. 1:**
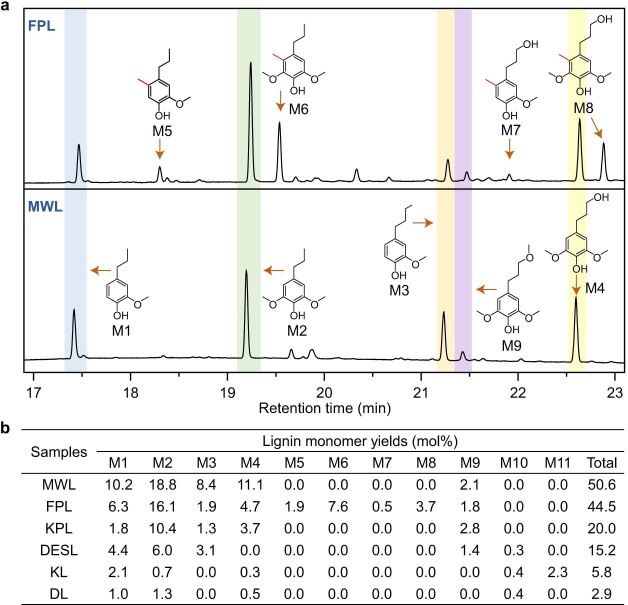
Hydrogenolysis of lignins to monomers. **a**, GC spectra of lignin monomers from hydrogenolysis of lignins. **b**, Yields of monomers resultant from hydrogenolysis of lignin samples that were extracted with different methods. MWL: Milled wood lignin; FPL: formaldehyde-protected lignin; KPL: acetone-protected lignin; DESL: deep eutectic solvent-extracted lignin; KL: kraft lignin; DL: dioxane-HCl lignin. M1: 2-methoxy-4-propylphenol; M2: 2,6-dimethoxy-4-propylphenol; M3: 4-(3-hydroxypropyl)-2-methoxyphenol; M4: 4-(3-hydroxypropyl)-2,6-dimethoxyphenol; M5: methylated propylguaiacol (2-methoxy-5-methyl-4-propylphenol); M6: methylated propylsyringol (2,6-dimethoxy-3-methyl-4-propylphenol); M7: methylated propanolguaiacol [4-(3-hydroxypropyl)-2-methoxy-5-methylphenol]; M8: methylated propanolsyringol [4-(3-hydroxypropyl)-2,6-dimethoxy-3-methylphenol]; M9: 2,6-dimethoxy-4-(3-methoxypropyl)phenol; M10: 4-ethyl-2,6-dimethoxyphenol; M11: 2,6-dimethoxyphenol.

**Extended Data Fig. 2:**
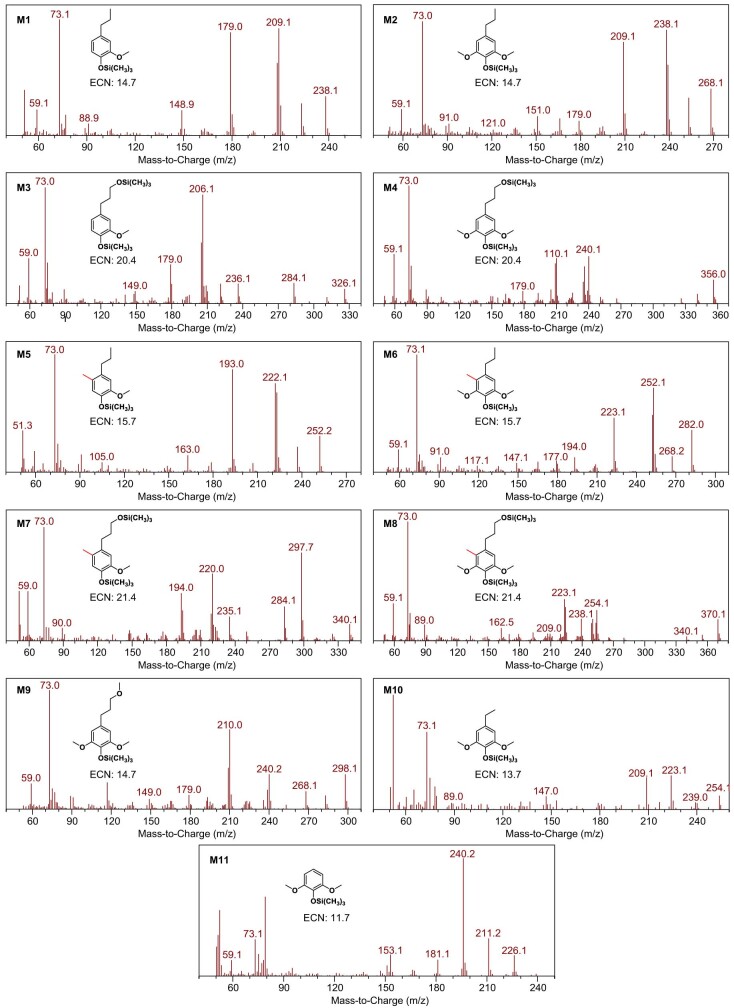
Mass spectra of lignin monomers from hydrogenolysis of lignins. All monomer samples were silylated by N, O-bis(trimethylsilyl)trifluoroacetamide (BSTFA) prior to gas chromatography-mass spectrometry (GC-MS) analysis. See ‘Lignin characterizations’ in the Method section for details.

**Extended Data Fig. 3:**
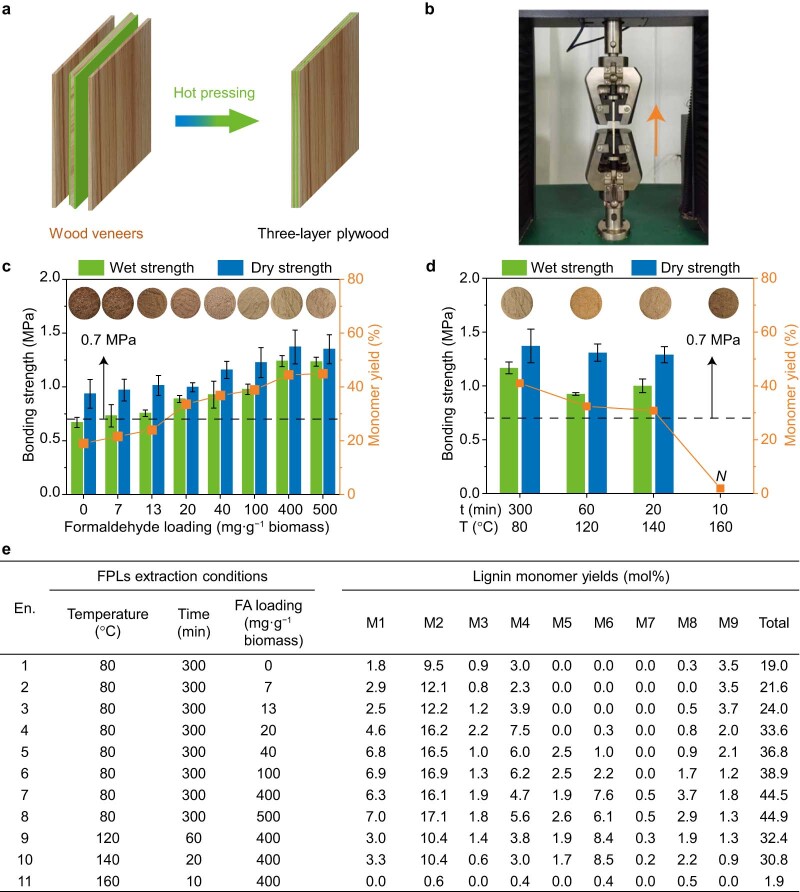
Comparison of adhesion performance of FPLs with different condensation degrees. **a**, Schematic illustration of the preparation process of three-layer plywood. Each layer was oriented with its grain perpendicular to that of the adjoining layer. **b**, The photograph of a bonding strength test. **c**, **d**, Effects of formaldehyde loadings (c) and extraction temperatures and times (d) on the condensation degree and adhesion performance of FPLs. Extraction temperature and time are the reaction temperature and time required for cooking biomass in a reactor to isolate lignin from biomass. At a specific formaldehyde loading, low extraction temperature or short extraction time facilitate to reduce the condensation of isolated FPLs and improve their adhesion performance. A higher temperature (140 vs. 80 °C) facilitated FPL extraction in shorter time (20 vs. 300 min), meanwhile maintaining the relatively low condensation degree. Although the wet strength of FPL-bonded plywood went down from 1.17 ± 0.05 MPa (mean ± standard deviation; 80 °C, 300 min) to 1.00 ± 0.06 MPa (mean ± standard deviation; 140 °C, 20 min), which was still higher than 0.7 MPa. In contrast, FPL that was extracted at 160 °C for 10 min had a monomer yield of only 1.9%, showed no adhesion. **e**, Yields of monomers resultant from hydrogenolysis of FPLs isolated under different conditions. The structures of silylated lignin monomers (M1–M9) were given in Extended Data Fig. 2. The hot-pressing conditions of lignin adhesives used to prepare plywoods in c and d were 190 °C (hot-pressing temperature), 8 min (hot-pressing time), 1.5 MPa (hot-pressing pressure), and 100 g glue·m^−2^ (glue application level).

To validate our assumption that uncondensed or slightly condensed lignins would perform well as adhesives, lignins separated from eucalyptus wood particles with different methods (milled wood lignin (MWL), formaldehyde-protected lignin (FPL), acetone-protected lignin, deep eutectic solvent-extracted lignin, kraft lignin and dioxane–HCl lignin) were investigated as adhesives (Methods) by simply mixing them with deionized water to form suspensions. These lignins had different condensation degrees, as reflected, inversely, by the molar yields of resultant aromatic monomers from their hydrogenolysis (Extended Data Figs. 1 and 2). The adhesion performance of the adhesives from each of these lignins was evaluated by measuring the dry (for applications in dry environments) and wet (for applications in humid environments) adhesion strengths of three-layer plywoods prepared with them (Fig. 1b and Extended Data Fig. 3a,b). MWL, FPL and acetone-protected lignin adhesives that were prepared with lignins separated under either mild conditions or protection by aldehyde or ketone demonstrated reasonable bonding strengths after hot pressing at 190 °C and 1.5 MPa for 8 min; both the dry and wet adhesion strengths met the minimum requirement of 0.7 MPa (Chinese National Standard GB/T 9846-2015; Fig. 1c), and wood failure (Fig. 1b) was observed. By contrast, three-layer plywoods prepared from deep eutectic solvent-extracted lignin, kraft lignin and dioxane–HCl lignin adhesives failed the dry and wet strength tests. Kraft lignin and dioxane–HCl lignin adhesives demonstrated essentially no adhesion property for wood veneers. The bonding strengths were found to be highly dependent on the condensation degrees of isolated lignins. MWL and FPL, which had hydrogenolysis monomer yields of 50.6% and 44.5%, were slightly condensed, resulting in the remarkable bonding strengths; by contrast, kraft lignin and dioxane–HCl lignin, which had monomer yields of only 5.8% and 2.9% (Fig. 1c and Extended Data Fig. 1), were severely condensed and did not show any adhesion property following hot pressing. This initial study revealed that slightly condensed or protected lignins from different sources could be directly used as wood adhesives without additional physical or chemical treatments.

**Extended Data Fig. 4:**
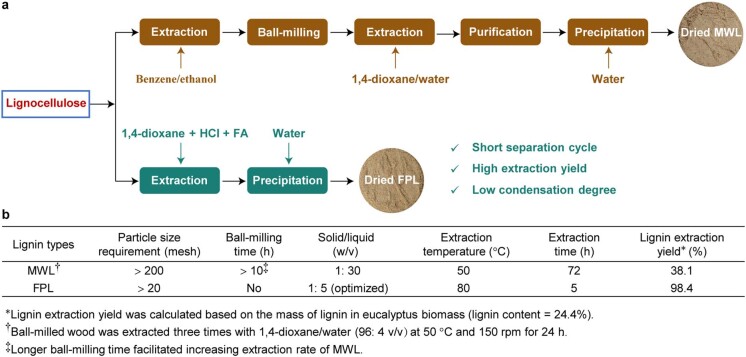
Comparisons of MWL and FPL isolation processes. **a**, The procedures of isolating MWL and FPL. **b**, Comparisons of isolation conditions. The MWL isolation method required time-consuming milling of wood into fine power and repeated solvent extractions, making it is an extremely energy-intensive and high-cost process. The FPL isolation method used larger particles, much less solvents, and shorter separation time, and yielded more lignin.

Despite the good adhesion performance of MWL, large-scale isolation of MWL is not industrially practical owing to energy-intensive milling, long separation time and low extraction yield^15,16^ (Extended Data Fig. 4). FPL was chosen for further study because of its high scalability and regulable condensation degrees by varying formaldehyde loading (Fig. 1d and Extended Data Fig. 3c) and other isolation conditions (for example, temperature and time) during extraction (Extended Data Fig. 3d). With formaldehyde loading increasing from 0 to 400 mg per gram of biomass, the monomer yield resulting from hydrogenolysis of FPLs gradually increased from 19.0% to 44.9% (Fig. 1d and Extended Data Fig. 3e), inversely correlating with the decreasing condensation degree of FPLs. At a hot-pressing temperature of 170 °C, wet and dry strengths of three-layer plywoods prepared with these FPLs as adhesives increased from 0.32 ± 0.10 to 0.99 ± 0.18 MPa and 0.69 ± 0.10 to 1.28 ± 0.17 MPa, respectively (Fig. 1d). Further increasing the formaldehyde loading from 400 to 500 mg per gram of biomass had negligible impact on the adhesion performance of FPLs owing to already completed protection of lignin from condensation (Fig. 1d). At a higher hot-pressing temperature (190 °C), wet and dry strengths of three-layer plywoods prepared with more condensed FPLs could be improved to meet the minimum industrial requirement (0.7 MPa; Extended Data Fig. 3c). At a specific formaldehyde loading, a low extraction temperature facilitated reduction of the condensation of isolated FPLs and improved their adhesion performance (Extended Data Fig. 3d). These comparative studies further confirm that the adhesion performance of lignin adhesives is highly inversely dependent on the condensation degree of lignins and that retaining at least a moderate fraction of the original uncondensed structure enables self-crosslinking of lignins during hot pressing.

**Fig. 2:**
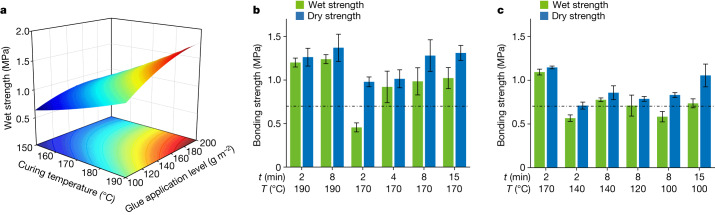
Effects of hot-pressing conditions on adhesion performances of lignin adhesives. **a**, Response surface for the wet strengths of three-layer plywoods as a function of the hot-pressing temperature and the glue application level. **b**, Effects of hot-pressing temperatures and times on the adhesion performance of FPL adhesives, without acid addition. Lignin adhesive preparation: 1:2 (w/w) lignin/water; glue application level: 100 g m^−2^. **c**, The promoting effect of acid addition on the adhesion performances of FPL adhesives. Lignin adhesive preparation: 1:2:0.1 (w/w/w) lignin/water/H_2_SO_4_; glue application level: 100 g m^−2^. The dot-dashed line at 0.7 MPa in **b**,**c** marks the minimum industrial requirement for the adhesion strength. All examined bonding strengths were found to be significantly (ANOVA, *P* < 0.01) affected by hot-pressing temperatures and time as well as glue application levels (Extended Data Table 1). Error bars show the s.d. of measured bonding strengths with four repeats.

Energy efficiency and productivity are the crucial consideration for plywood manufacturers. Although the strengths of FPL-bonded plywoods could be gradually improved by increases in the hot-pressing temperature and time as well as the glue application level (Fig. 2a,b), these processing conditions were energy-intensive and time-consuming. For the consideration of cost and industrial applicability, the adhesion performance of FPL adhesives was further optimized. As shown in Fig. 2b, when the hot-pressing temperature was reduced from 190 to 170 °C, the wet strength of FPL-based plywoods prepared with a hot-pressing time of 2 min was decreased from 1.20 ± 0.05 to 0.46 ± 0.05 MPa but it could be gradually improved from 0.46 ± 0.05 to 1.00 ± 0.12 MPa by prolonging the hot-pressing time from 2 to 15 min, indicating that prolonging the hot-pressing time enables a lower hot-pressing temperature. The addition of sulfuric acid as a crosslinking catalyst substantially reduced the hot-pressing time from 15 to 2 min and the hot-pressing temperature from 170 to 100 °C (Fig. 2b,c), consistent with the general knowledge that an acidic catalyst could promote lignin condensation. An acidic curing condition therefore facilitates lignin self-crosslinking at moderate temperatures and/or in a short time, saving the time and energy required for making plywood.

**Fig. 3:**
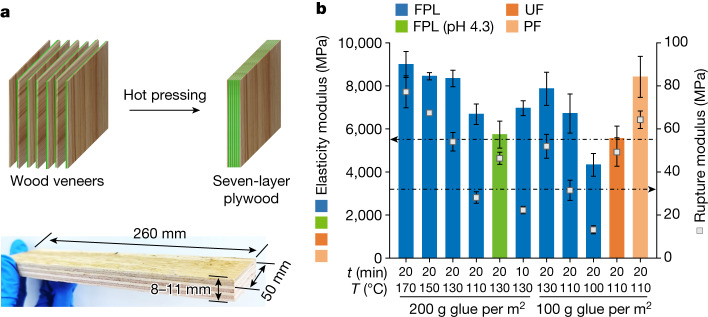
Mechanical properties of seven-layer plywoods prepared from lignin adhesives. **a**, Schematic illustration of preparation (top) of seven-layer plywood specimens for mechanical performance tests (bottom). **b**, The MOEs and MORs of seven-layer plywoods bonded with FPL, urea–formaldehyde and phenol–formaldehyde resin adhesives. The dot-dashed lines at 5,500 MPa and 32 MPa mark the minimum industrial requirements for the MOE and MOR, respectively. Lignin adhesive preparation: 1:4 (w/w) lignin (or urea–formaldehyde or phenol–formaldehyde)/water 1:4, pH 2.1 (except as indicated); hot-pressing pressure: 2.0 MPa. All examined moduli were found to be significantly (ANOVA, *P* < 0.01) affected by hot-pressing temperature, time and pH but not the glue application level (Extended Data Table 1). Error bars show the s.d. of measured bonding strengths with four repeats.

**Extended Data Fig. 5:**
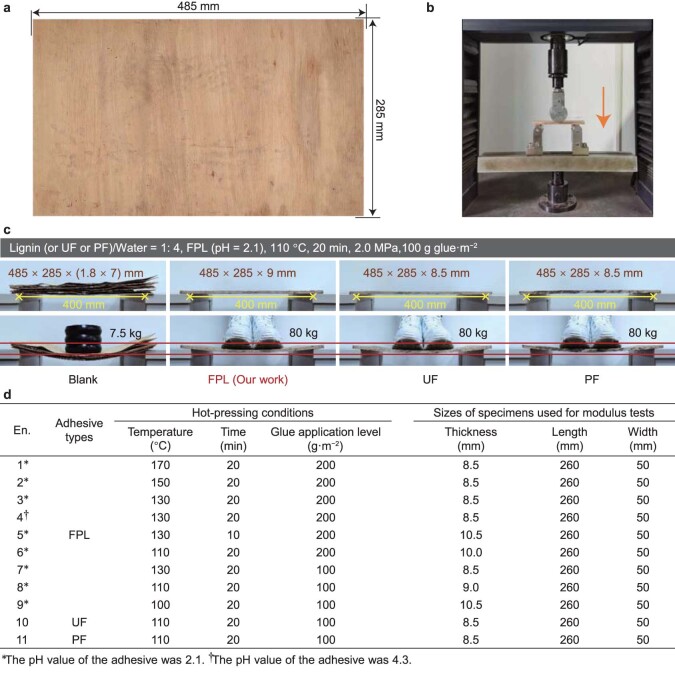
Mechanical performance tests of seven-layer plywood. **a**, The photograph of a whole piece of prepared seven-layer plywood. **b**, Photograph of elasticity and rupture modulus tests. **c**, Photographs of piled veneers or plywoods before (top row) and after (bottom row) 7.5-Kg or 80-Kg loading. The order of the deformation degree after the 80-Kg loading was UF resin > FPL ≈ PF resin, showing the excellent mechanical properties of FPL adhesive. **d**, The dimensions of seven-layer plywoods prepared at different hot-pressing conditions. The FPL used in this study was isolated from eucalyptus at 80 °C (extraction temperature), 5 h (extraction time), and 400 mg formaldehyde·g^−1^ biomass. The mass ratio of lignin, UF or PF resin to deionized water in prepared adhesive suspensions was 1: 4 (w: w). All plywoods in d were prepared under the hot-pressing conditions of 110 °C (hot-pressing temperature), 20 min (hot-pressing time), 2.0 MPa (hot-pressing pressure), and 100 g·m^−2^ (glue application level).

Multilayer plywood products have broader applications than the three-layer plywood, but they require longer hot-pressing time owing to heat transfer limitation. The modulus of elasticity (MOE) and the modulus of rupture (MOR) are important mechanical parameters for multilayer plywoods (Fig. 3a and Extended Data Fig. 5a,b). The results in Fig. 3b show that the MOE and MOR of all seven-layer plywoods prepared at 110–170 °C for 20 min met the minimum requirements (Chinese National Standard GB/T 9846-2015; MOE, 5,500 MPa; MOR, 32 MPa) with a glue application level of 200 g m^−2^. As the glue application level was reduced to 100 g m^−2^, a qualified MOE (6,716 ± 907 MPa) and MOR (32 ± 4 MPa) could also be achieved at 110 °C for 20 min. However, these two glue application levels did not have significant effects on the MOE and MOR (Extended Data Table 1). Compared to seven-layer plywoods bonded with phenol–formaldehyde and urea–formaldehyde resin adhesives, those bonded with FPL adhesives showed comparable MOEs and MORs under the same curing conditions (Fig. 3b and Extended Data Fig. 5c). When the pH of FPL adhesives was adjusted from 2.1 to 4.3, the MOE of the corresponding plywood (highlighted in green in Fig. 3b) was substantially reduced from 8,344 ± 382 to 5,736 ± 629 MPa at 130 °C and 1.5 MPa for 20 min, further confirming that a lower pH facilitates the crosslinking of uncondensed lignins during hot pressing and thereby better adhesion performance.

**Fig. 4:**
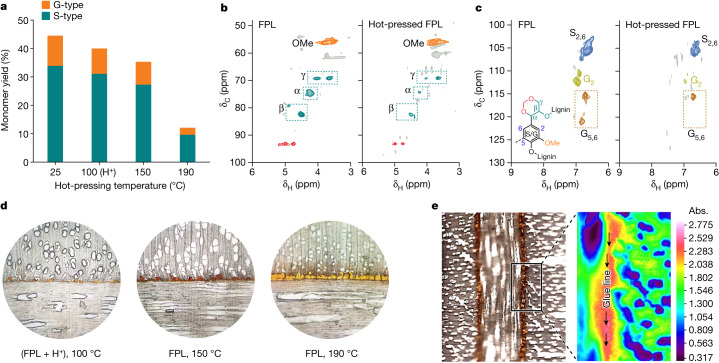
Adhesion mechanism of lignin adhesives for bonding wood. **a**, Yields of resultant aromatic monomers from hydrogenolysis of FPLs before (25 °C) and after hot pressing. The H^+^ shown in **a**,**d** indicates the addition of sulfuric acid as a crosslinking catalyst to lignin adhesives. **b**,**c**, Side-chain (**b**) and aromatic (**c**) regions in heteronuclear single quantum coherence spectra of FPL before and after hot pressing at 190 °C (δ, chemical shift; the chemical structure in the left panel of **c** represents an acetal-protected lignin monomeric unit). **d**, Optical microscopy images of the glue lines in the plywood products prepared at different hot-pressing temperatures. **e**, Scanning electron microscopy (left) and Fourier transform infrared (FTIR) microscopy (right) images of the glue-line region in the plywood prepared at 190 °C (the FTIR image was recorded at 1,597 cm^−1^, a characteristic absorption peak of lignin; Extended Data Fig. 8f). See Methods for details of sample preparation and characterization.

**Extended Data Fig. 6:**
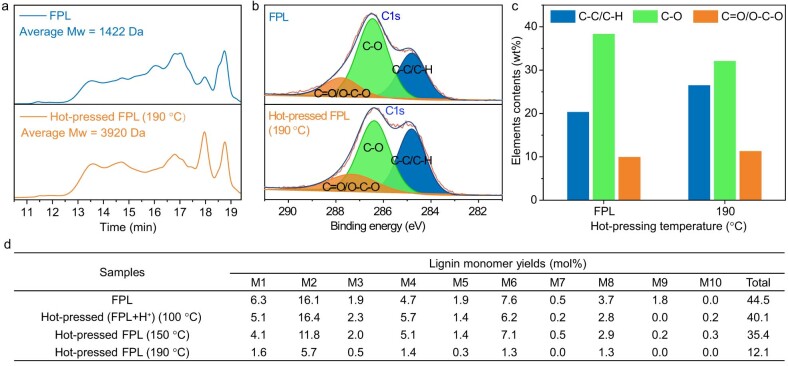
Characterization of FPLs before and after hot-pressing. **a**, GPC spectra and molecular weights of hydrogenolysis products from FPLs before and after hot-pressing. **b**, **c**, C1s in XPS spectra (b) and the contents of carbon-based linkages (c). **d**, Hydorgenolysis monomer yields of FPL and hot-pressed FPLs. The hot-pressed lignins were prepared by hot-pressing FPLs wrapped by aluminum foil. Hot-pressing conditions: lignin/water = 1: 2 (w: w), 190 °C (hot-pressing temperature), 8 min (hot-pressing time), pH = 7, and 1.5 MPa (hot-pressing pressure). Increased C–C and decreased C–O contents were observed after hot-pressing FPLs at 190 °C, indicating that a high temperature facilitated the occurrence of condensation or the formation of additional C–C linkages in FPLs during hot-pressing. The structures of silylated lignin monomers (M1–M9) were given in Extended Data Fig. 2.

**Extended Data Fig. 7:**
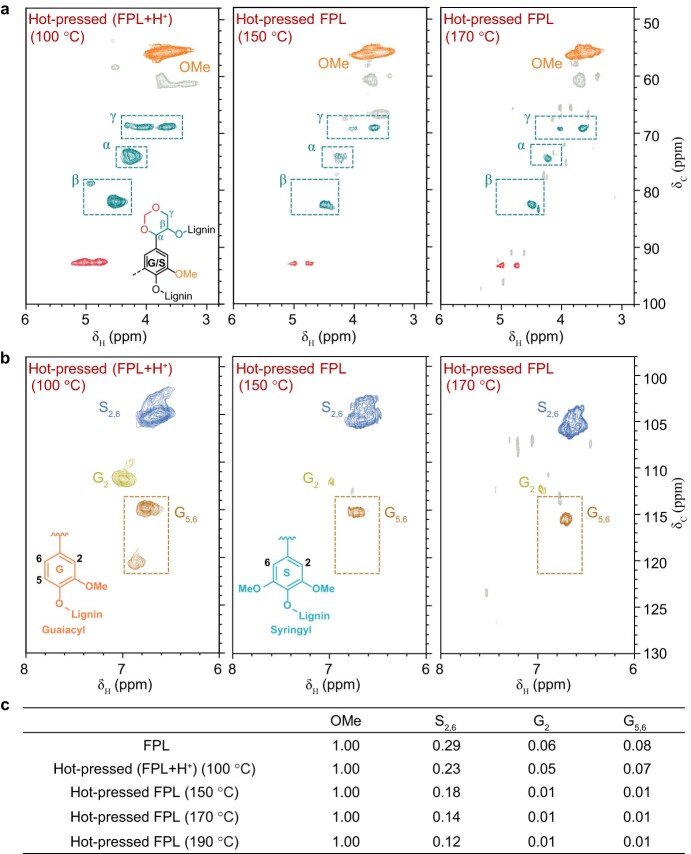
HSQC spectra of FPL before and after hot-pressing. **a**, **b**, Side-chain (a) and aromatic (b) regions in HSQC spectra of FPL before and after hot-pressing. **c**, Contents of the vacant sites on the aromatic nuclei of FPL before and after hot-pressing determined by HSQC. The hot-pressed lignins were prepared by hot-pressing FPL that were wrapped by aluminum foil under different temperatures; other hot-pressing conditions: lignin/water = 1: 2 (w: w), 8 min, pH = 7, and 1.5 MPa.

The occurrence of lignin self-crosslinking could be confirmed with the increased presence of C–C linkages in hot-pressed lignins, which was implied by decreased monomer yields and increased molecular weights of the resultant products from hydrogenolysis of hot-pressed FPL lignins as well as increased C–C and decreased C–O contents in hot-pressed FPL lignins (Fig. 4a and Extended Data Fig. 6). In addition, a comparison of the heteronuclear single quantum coherence spectra of FPLs before and after hot pressing showed diminished H signals of side chains and particularly aromatic nuclei of the hot-pressed FPLs (for example, G5 and G6; Fig. 4b,c and Extended Data Fig. 7), implying that both the side chain and the aromatic nuclei participated in the crosslinking of lignin adhesives.

**Extended Data Fig. 8:**
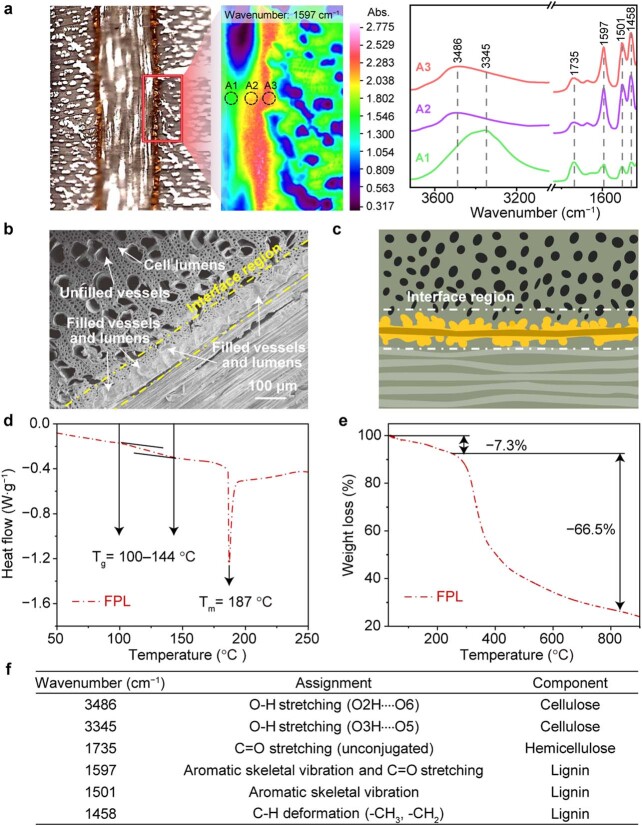
Interfacial analysis of the glueline region and thermal analysis of FPL. **a**, FTIR microscopy image of the glueline region recorded at a wavenumber of 1597 cm^−1^. **b**, **c**, SEM image (b) and schematic illustration (c) of the glue line region. These images demonstrated that the vessels at the glueline region were filled with lignin adhesives after hot pressing. **d**, **e**, DSC (d) and TG results (e) of FPL. Due to the relatively low glass transition temperature of FPL (T_g_ = 100–144 °C), FPL adhesives could be softened at a low hot-pressing temperature as low as 100 °C. **f**, The characteristic linkages in biomass and their corresponding FTIR absorption wavenumbers.

With optical microscopy and Fourier transform infrared microscopy, the filling of vessels with lignin adhesives could be observed under three hot-pressing temperatures (100, 150 and 190 °C; Fig. 4d,e and Extended Data Fig. 8a–c). The filling of vessels was very limited at 100 °C because of the low flowability of FPL (Extended Data Fig. 8d,e), but it was increased with the hot-pressing temperature increasing from 100 to 190 °C (Fig. 4d). Despite limited mechanical interlocking, the plywood prepared at 100 °C still showed good adhesion performance (Fig. 2c) probably due to the interfacial crosslinking between the lignins in the adhesives and the cell wall. Nevertheless, the possible interfacial bonding and penetration of lignin adhesives into cells require further validation in the future. On the basis of these observations, we infer that such lignin adhesives would have an adhesion mechanism involving softening of lignin by water, filling of vessels with softened lignin and crosslinking of lignins in adhesives and the cell wall.

**Extended Data Fig. 9:**
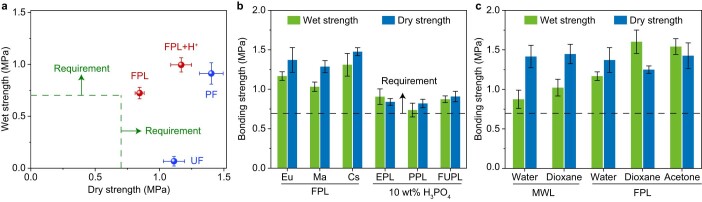
Comparison of UF and PF resins as well as a variety of lignin adhesives in adhesion performance. **a**, Comparison of adhesion performances of FPL adhesives and conventional PF and UF resin adhesives. Lignin adhesive preparation in a lignin (or UF or PF)/water = 1: 2 (w: w); hot-pressing conditions: 150 °C, 8 min, 1.5 MPa, and a glue application level of 100 g·m^−2^. Compared to the three-layer plywoods bonded with PF and UF resin adhesives, FPL adhesive showed comparable adhesion strength. **b**, Lignin adhesives prepared from different feedstocks protected with formaldehyde as well as eucalyptus lignin protected with other aldehydes. Eu: eucalyptus; Ma: Masson pine; Cs: corn stover; FPL: formaldehyde-protected lignin; EPL: ethyl aldehyde-protected lignin; PPL: propionaldehyde-protected lignin; FUPL: furfural-protected lignin; MWL, milled wood lignin. The H_3_PO_4_ loading was on the basis of lignin. **c**, Lignin adhesives prepared via mixing FPL with different solvents. Lignin adhesive preparation in b and c: lignin/water (or solvent) = 1: 2 (w: w), pH = 7; hot-pressing conditions in b and c: 190 °C, 8 min, 1.5 MPa, and a glue application level of 100 g·m^−2^.

**Extended Data Fig. 10:**
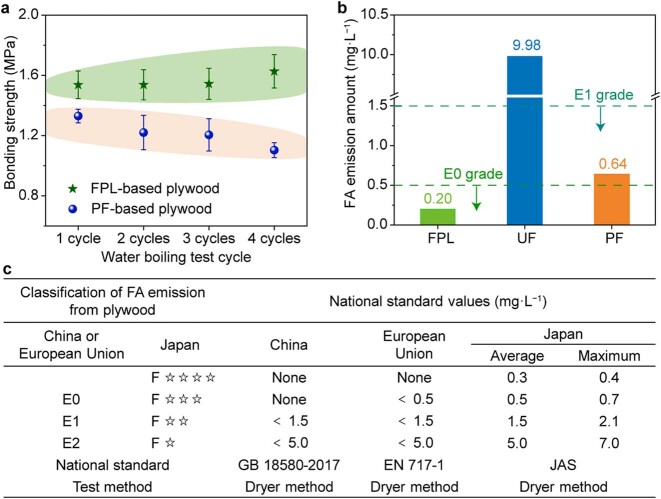
Comparison of UF and PF resins as well as a variety of lignin adhesives in weather resistance and formaldehyde emission. **a**, Weather resistance. The UF-based three-layer plywoods failed the boiling water test at the first cycle as expected, suggesting the poor weather-resistance of UF resin adhesive. As the test cycle increased, the bonding strength of PF-based three-layer plywoods decreased gradually from 1.33 ± 0.05 (mean ± standard deviation) to 1.10 ± 0.05 (mean ± standard deviation) MPa while that of FPL-based plywoods slightly increased from 1.53 ± 0.09 (mean ± standard deviation) to 1.63 ± 0.11 (mean ± standard deviation) MPa. Since the synthesis and curing of PF resins are generally performed under an aqueous alkaline condition, part of the PF resin may be dissolved in water during the water boiling test, thereby leading to the gradual decrease of the strength. Different from PF resins, FPL was water-insoluble and the curing of FPL could continuously proceed in a neutral or acidic environment (e.g., the boiling water), thereby leading to the gradually increased strengths. **b**, Formaldehyde emission amount of three-layer plywoods prepared with FPL adhesives and UF and PF adhesives. **c**, Comparison of different national standards for classifying the formaldehyde emission amounts from plywoods. The test report from a specialty agent showed that the formaldehyde emission amount from the plywood bonded with UF resin was 9.98 mg∙L^−1^, which did even not meet the E2 grade of Chinese National Standard GB/T 18580-2017. The formaldehyde emission amounts of the plywoods bonded with PF resin and FPL adhesive were 0.64 and 0.2 mg∙L^−1^, respectively, meeting the E1 grade (≤ 1.5 mg∙L^−1^) and the E0 grade (≤ 0.5 mg∙L^−1^) of European standard (EN 717-1). The comparison indicates that FPL adhesive is a highly environment-friendly bio-based adhesive and safer than the traditional formaldehyde-containing wood adhesives.

In the above study, lignin adhesives prepared from the mixture of FPL and water demonstrated remarkable mechanical performance and tunable processability comparable to those of traditional urea–formaldehyde and phenol–formaldehyde resin adhesives (Extended Data Fig. 9a). We further found that a variety of FPL adhesives prepared with organic solvents (for example, dioxane and acetone) as mixing solvents and from different feedstocks (for example, Masson pine (a softwood) and corn stover (an agricultural residue)) as well as lignin adhesives prepared from lignins protected with other aldehydes (for example, acetaldehyde, propionaldehyde, and furfural^17,18^) showed qualified adhesion performances (>0.7 MPa; Extended Data Fig. 9b,c). Besides, plywood prepared from FPLs was able to survive the weather-resistance test, indicating its potential to be used in humid or exterior environments (Extended Data Fig. 10a). Its low formaldehyde emission level (Extended Data Fig. 10b,c) may also afford its application in an interior environment. These superior properties make uncondensed or slightly condensed lignins such as acetal-protected lignins promising alternatives to traditional petrochemical-based wood adhesives in preparing not just plywood but also other pressed wood products such as particleboards and fibreboards.

## Methods

### Materials and chemicals

All commercial chemicals were analytical reagents, and were used without further purification. The following were purchased from Aladdin Chemicals: 1,4-dioxane (>99%), formaldehyde (37 wt% in water), lactic acid (85–90%), choline chloride (ChCl, 98%), NaOH (>98%), phosphoric acid (85–90 wt% in water), acetaldehyde (99%), propionaldehyde (97%), furfural (99%), hexadecane (>99.5%), dodecane (>99.7%), methyl isobutyl ketone (99.5%), urea–formaldehyde resin (solid content: 60%), phenol–formaldehyde resin (solid form), methanol (99.5%), 5% Ru on carbon (wetted with about 50% water), pyridine (anhydrous, 99.8%), *N*,*O*-bis(trimethylsilyl)trifluoroacetamide (97% BSTFA, containing 1% trimethylchlorosilane), chloroform-d (99.96 atom% D, containing 0.03% (v/v) tetramethylsilane) and tetrahydrofuran (>99%). Acetone (>99%), HCl (37%) and sulfuric acid (98%) were purchased from Sinopharm.

Corn stover was supplied by the State Key Laboratory of Biobased Materials and Green Papermaking at Qilu University of Technology, China. Eucalyptus and Masson pine wood chips were provided by Fujian Qingshan Paper Co. Before experiments, corn stover and wood chips were milled to pass through a 0.85-mm screen. Kraft lignin was obtained directly from Longlive.

### Lignin extraction

Several representative lignins were chosen for preparing lignin adhesives. The methods for extracting these lignins were as follows.

#### MWL

MWL was isolated from eucalyptus according to a previously reported method^16^ (Extended Data Fig. 4). Specifically, 5 g of air-dried eucalyptus wood particles (>20 mesh) was first extracted with 150 ml of benzene–ethanol (2:1, v/v) for 24 h in a Soxhlet extractor to remove extractives. The extractive-free eucalyptus wood particles were subjected to milling with ZrO_2_ balls at 600 r.p.m. for 10 h in a planetary ball mill (Pulverisette 7 Premium Line, Fritsch). A 20 g quantity of the ball-milled wood was extracted three times with 200 ml 96% dioxane–water (96:4, v/v) at 50 °C and 150 r.p.m. for 24 h. All extracts were combined and concentrated at 50 °C with a vacuum evaporator. The concentrated lignin solution (around 20 ml) was dried by freeze-drying to obtain crude MWL, which was further purified by dissolution in 20 ml 90% acetic acid/water (v/v) followed by precipitation in 200 ml deionized water. The purified MWL was collected by centrifugation, followed by washing with water until the pH of the filtrate reached neutral and freeze-drying under vacuum. The obtained dry MWL was directly used for preparing lignin adhesives by simply mixing it with deionized water at room temperature.

#### FPL

The extraction of FPL was carried out according to our previously reported lignin protection strategy^19^. FPLs with different condensation degrees were isolated through varying formaldehyde loadings from 0 to 500 mg per gram of biomass and extraction temperature and time. Specifically, 500 g of air-dried wood particles (passed through 20 mesh), 4.5 l of 1,4-dioxane, 96–685 ml of a formaldehyde aqueous solution (37 wt%; that is, 70–500 mg formaldehyde per gram of biomass) and 210 ml of HCl solution (37 wt%) were loaded into a 10-l glass reactor equipped with a mechanical stirrer. The reactor was heated to a specified temperature (80–160 °C) in an oil bath and maintained at a specific temperature for a certain time (10–300 min). The reaction was stirred at 300 r.p.m. using a mechanical stirrer. After the reaction, the reactor was cooled to room temperature. The acidic slurry was then neutralized by addition of a bicarbonate solution (210 g in 2.5 l water). The neutralized solution was filtered and the residue after filtration was washed with 1,4-dioxane until the filtrate was colourless. All filtrates were collected and divided into two equal parts (around 4 l × 2), which were slowly poured into two buckets filled with 30 l of deionized water to precipitate lignin. The FPL was collected by filtration, washed with deionized water until the pH of the filtrate reached neutral and then freeze-dried under vacuum. The obtained dry FPLs were directly used for preparing lignin adhesives by simply mixing them with deionized water to form suspensions at room temperature. All FPLs except for those used in Fig. 1d and Extended Data Fig. 3c,d were isolated with a formaldehyde loading of 400 mg per gram of biomass at 80 °C for 5 h. FPLs used in Fig. 1d and Extended Data Fig. 3c were isolated with different formaldehyde loadings at 80 °C for 5 h. FPLs used in Extended Data Fig. 3d were isolated with a formaldehyde loading of 400 mg per gram of biomass under different extraction temperatures and times.

For preparation of acidic FPL adhesives, all procedures used here were the same as the procedures used for preparing dry FPLs, except the residue (precipitated lignin) was washed with deionized water until the pH of the filtrate was 2.1. The obtained FPL paste (without drying) was used to prepare lignin adhesives with the addition of deionized water.

#### Acetone-protected lignin

The extraction procedures for acetone-protected lignin were the same as that for FPL, except that formaldehyde solution was replaced by 500 ml acetone (that is, 1 g acetone per gram of biomass)^17^. The acetone-protected lignin used in this study was prepared with an extraction temperature of 80 °C and an extraction time of 5 h.

#### Deep eutectic solvent-extracted lignin

The deep eutectic solvent^20^ was prepared by mixing lactic acid with choline chloride at a molar ratio of 2:1, followed by melting at 60 °C for 3 h in a vacuum oven. The solid mixture was stirred periodically until a homogeneous and transparent liquid was obtained. Once the liquid was formed, the mixture was cooled in a desiccator to room temperature to avoid moisture absorption. The extraction of lignin was carried out in a 350-ml pressure-resistant flask. The prepared deep eutectic solvent (250 g) and 25 g of air-dried wood particles (passed through 20 mesh) were loaded into the flask. The mixture was heated to 100 °C and stirred at 300 r.p.m. with a stir bar at the same temperature for 6 h. The flask was then cooled to room temperature. The mixture was filtered, and the residue after filtration was washed with deionized water until the filtrate was colourless. All of the filtrates (around 300 ml) were collected and slowly poured into a beaker filled with 4 l of deionized water to precipitate lignin. The deep eutectic solvent-extracted lignin was collected by filtration, washed with deionized water until the pH of the filtrate reached neutral, and freeze-dried under vacuum. The obtained dry deep eutectic solvent-extracted lignin was used to prepare lignin adhesives by simply mixing them with deionized water at room temperature.

#### Dioxane–HCl lignin

The extraction procedures for dioxane–HCl lignin were the same as that for FPL, except that the formaldehyde solution was not added and was replaced by 500 ml deionized water^19^. The dioxane–HCl lignin used in this study was prepared with an extraction temperature of 140 °C and an extraction time of 20 min.

#### Other acetal-protected lignins

The extraction procedures for other acetal-protected lignins such as acetaldehyde-, propionaldehyde- and furfural-protected lignins were the same as that for FPL, except that formaldehyde solution was replaced by 500 ml of the corresponding aldehydes (that is, 1 g aldehyde per gram of biomass)^17^. All of these acetal-protected lignins used in this study were prepared with an extraction temperature of 80 °C and an extraction time of 5 h.

### Preparation of lignin adhesives

For making three-layer plywood, lignin adhesives were prepared by simply mixing dry lignin powder (pH = 7) with solvents (water, 1,4-dioxane or acetone) at a weight ratio of 1:2 (w:w).

For making seven-layer plywood, lignin adhesives were prepared by supplementing an appropriate amount of deionized water to the wet lignin paste (pH 2.1) to give a lignin/water ratio of 1:4 (w/w).

### Preparation of plywoods

For preparing three-layer plywood, three poplar veneers (145 × 110 × 1.5 mm) were used, and each layer was oriented with its grain perpendicular to that of the adjoining layers. The adhesives were evenly applied on the two sides of the middle-layer veneer (Fig. 1b and Extended Data Fig. 3a) with a glue application level of 70–250 g m^−2^ on each side. Resultant veneers were combined and placed on the heating plate of a hot presser and hot pressed at 1.5–2.0 MPa and 100–190 °C for 2–15 min to obtain the three-layer plywood.

For preparing seven-layer plywood, seven eucalyptus veneers (485 × 285 × 1.8 mm) were used, and each layer was oriented with its grain perpendicular to that of the adjoining layer. The adhesives were evenly applied on the same single side of six veneers (Fig. 3a) with a glue application level of 100 or 200 g m^−2^ on each side. Six adhesive-coated veneers were combined in order and covered with the remaining (seventh) veneer on the top. The combined seven-layer veneers were hot pressed at 2.0 MPa and 100–170 °C for 10–20 min to obtain the seven-layer plywood. See Extended Data Fig. 5d for the thicknesses of seven-layer plywoods prepared under different conditions.

### Mechanical strength tests of plywoods

Bonding strengths (including dry and wet strengths) of three-layer plywood and the MOE and the MOR of seven-layer plywood were tested according to Chinese National Standards (GB/T 17657-2013) on an Instron 5967 universal testing machine. The specific sizes of three-layer plywood specimens used for bonding strength tests are presented in Fig. 1b. For a wet strength test of type II plywood (GB/T 17657-2013, for applications in humid environments), the specimens were pre-soaked in water at 63 ± 3 °C for 3 h and then air-dried at room temperature for 10 min before the test. The specific sizes of seven-layer plywood specimens used for mechanical strength tests are presented in Fig. 3a and Extended Data Fig. 5e.

### Weather (or boiling water)-resistance test

The weather resistance of plywoods prepared with FPL, urea–formaldehyde and phenol–formaldehyde adhesives was preliminarily evaluated by a boiling water test according to Chinese National Standard GB/T 17657-2013. The three-layer plywood specimens used for boiling water tests were the same as those used for bonding strength tests. The boiling water test was conducted in varied cycles to evaluate the water resistance of prepared three-layer plywoods. A cycle of the boiling water test was conducted according to the following procedure. The specimens were subjected to cooking in boiling water for 4 h, air-dried at 60 ± 3 °C for 20 h in an oven, and then stored at 4 °C for 12 h in a refrigerator. After specified cycles of the boiling water test, treated specimens were cooked again in boiling water for 4 h and air-dried in an oven at 60 ± 3 °C for 20 h and then used for the bonding strength test.

### Formaldehyde emission test

The amount of formaldehyde emitted from plywood was detected by a desiccator method in accordance with Chinese National Standard (GB 17657-2013). The grades of plywood in the test reports were determined in accordance with the Chinese National Standard for formaldehyde emission of engineered wood and its products used as indoor decorative materials (GB 18580-2017; Extended Data Fig. 10c).

### Lignin characterization

#### Hydrogenolysis of lignin into lignin monomers (lignin condensation degree)

In a 25-ml high-pressure-resistant reactor, 200 mg of the extracted lignin sample was mixed with 10 ml methanol and 100 mg Ru/C catalyst^21^. Once the reactor was closed, it was purged three times with 0.2 MPa H_2_ and then pressurized with 4 MPa H_2_. The reaction was stirred with a magnetic bar and the reactor was heated with a heating jacket controlled by a PID temperature controller to 220 °C and maintained at the temperature for 5 h. After the reaction, the reactor was cooled to room temperature. About 30 mg of dodecane was directly added as an internal standard into the reactor and mixed with the slurry. One millilitre of the clear solution was sampled and centrifuged. The resultant supernatant was used for gas chromatography (GC), GC with mass spectrometry (MS) and gel permeation chromatography analyses.

#### Lignin monomer analysis by GC and GC–MS

Lignin monomers resulting from hydrogenolysis were initially identified by GC–MS and then quantified by GC^9^. Before the GC and GC–MS analyses, the sample was derived with BSTFA. Specifically, 20 μl of the solution containing dodecane (an internal standard) was directly sampled into a 2-ml GC vial, followed by the addition of 200 μl of pyridine and 500 μl of BSTFA. The mixed solution (0.7 ml) was heated in an oven at 80 °C for 1 h. The silylated products were identified by GC–MS (Techcomp) using a Scion 436C series GC instrument equipped with an HP5-MS capillary column (30 m × 0.45 mm) and a Scion 436-GC-SQ series MS detector. The injection temperature was 300 °C. The column temperature programme was: 50 °C (5 min), 10 °C min^−1^ to 300 °C, and 300 °C (5 min). The monomer products were directly identified according to the MS spectra (Extended Data Fig. 2). The identified products were further quantified by a GC instrument (Scion 436C series, Techcomp) equipped with an HP5 capillary column and a flame ionization detector (FID). The operation conditions were the same as those used for the GC–MS analysis.

For convenience, the quantification of monomer (Extended Data Figs. 1 and 2) was based on an internal standard (dodecane) and the effective carbon number (ECN) method according to a previous report^19^ (Extended Data Fig. 2). The detailed yield calculations were as follows:

1 $${n}_{{\rm{product}}}={n}_{{\rm{dodecane}}}\times \frac{{A}_{{\rm{product}}}}{{A}_{{\rm{dodecane}}}}\times \frac{{{\rm{ECN}}}_{{\rm{dodecane}}}}{{{\rm{ECN}}}_{{\rm{product}}}}$$

2 $${Y}_{{\rm{product}}}=\frac{{n}_{{\rm{product}}}}{{n}_{{\rm{theoretical}}}}$$

in which *n*_dodecane_ (mmol) is the molar amount of the internal standard (dodecane), *n*_product_ (mmol) is the molar amount of the lignin monomers, *A*_product_ is the peak area of monomers in the GC–FID chromatogram, *A*_dodecane_ is the peak area of dodecane in the GC–FID chromatogram, ECN_dodecane_ is the effective carbon number of dodecane, ECN_product_ is the effective carbon number of the lignin monomers, *Y*_product_ is the molar yield of monomers calculated on the basis of the molar amount of lignin monomeric units (*n*_theoretical_); an average molecular weight of 210 g mol^−1^ for lignin monomeric units was used for the calculations.

#### Gel permeation chromatography analysis

The molecular weights of lignins were characterized by a gel permeation chromatography instrument (Waters Breeze 2) equipped with a refractive index detector (Waters model 2414) and two Waters Styragel columns (models: HR 0.5 and HR 2; dimensions: 7.8 × 300 mm)^22^. Polystyrenes with different molecular weights were used as standard compounds. Tetrahydrofuran was used as the mobile phase at a flow rate of 1.0 ml min^−1^. The column compartment and the refractive index detector temperature were set at 40 °C. To facilitate dissolution of lignins in tetrahydrofuran, hydrogenolysis of lignins and hot-pressed lignins was conducted before gel permeation chromatography analysis.

#### X-ray photoelectron spectroscopy analysis

The contents of carbon-derived linkages (C–C/C–H, C–O, C=O/O–C–O) of lignin adhesive samples were determined by X-ray photoelectron spectroscopy (ESCALAB 250, Thermo-Fisher Scientific) equipped with a monochromatic Al-Kα X-ray source^23^. The X-ray photoelectron spectra were collected at 200–600 eV. Thelignin adhesive samples used for X-ray photoelectron spectroscopy analysis were prepared by directly hot pressing FPL adhesive that were wrapped by aluminium foil.

#### 2D NMR heteronuclear single quantum coherence characterization

NMR spectra were recorded on a Bruker AVANCE III HD 600 MHz spectrometer^24^. Lignin samples (80 mg) were dissolved in 0.5 ml dimethylsulfoxide (DMSO)-d_6_ in an NMR tube. Owing to poor solubility in DMSO-d_6_, all hot-pressed lignins were ball milled in advance at 500 r.p.m. for 1 h in a planetary ball mill (Pulverisette 7 Premium Line, Fritsch) to facilitate dissolution. The Bruker program hsqcedetgpsisp 2.3 was selected for heteronuclear single quantum coherence characterization. Heteronuclear single quantum coherence characterization of the lignin samples was carried out with the following parameters: acquired from 10 to 0 ppm in F2 (^1^H) with 2,048 data points and a 1-s recycle delay, 160 to 0 ppm in F1 (^13^C) with 256 increments of 64 scans. The total acquisition time for a sample was 5 h. The central DMSO solvent peak δ ppm (39.5, 2.49) was used for calibration of correlation peaks. The heteronuclear single quantum coherence spectra were analysed with MestReNova 6.1.0.

#### Differential scanning calorimetry and thermogravimetry analysis

The curing temperatures of lignin samples were analysed with a differential scanning calorimeter (DSC; Q2500, TA Instruments)^3^. Before the DSC analysis, lignin samples were freeze-dried to remove excess water. For DSC analysis, the freeze-dried lignin samples were placed in a high-volume aluminium crucible sealed with a lid, and then heated from 30 to 250 °C at a rate of 5 °C min^−1^ under a nitrogen atmosphere with a flow rate of 50 ml min^−1^.

Thermal stability of lignin samples was analysed with a thermogravimetry instrument that was operated at 10 °C min^−1^ from 30 to 900 °C under a nitrogen atmosphere with a flow rate of 50 ml min^−^^3^.

### Wood–adhesive interface characterizations

#### Optical microscopy analysis

The glue lines in plywood samples were observed on an optical microscope (Nicon E200MV). The three-layer plywood was cut into small pieces (10 × 5 × 3 mm) using a small table saw. Slices with a thickness of 15 μm were cut with a sliding microtome from the small pieces for optical microscopy analysis.

#### Fourier transform infrared microscopy analysis

The Fourier transform infrared microscopy analysis of plywood samples was conducted on a Spectrum Spotlight 400 FTIR microscope connected to a Spectrum FTIR Frontier spectrometer (Perkin Elmer) with a transmission mode^25^. Slices with a thickness of 24 μm were cut with a sliding microtome from the three-layer plywood specimens. Before cutting, the three-layer plywood specimens were softened by soaking them in hot water at 70 °C for 10 h to facilitate the preparation of transverse sections. Measurements provided a pixel resolution of 6.25 × 6.25 μm^2^. The Fourier transform infrared spectra were recorded from 4,000 to 720 cm^−1^ with a 6 μm^−1^ spectral resolution. The Fourier transform infrared microscopy imaging at a wavenumber of 1,597 cm^−1^  was conducted for the glue line region at a size of 1,100 × 700 μm^2^.

#### Scanning electron microscopy

The three-layer plywood was cut into small pieces (10 × 3 × 3 mm) using a small table saw, and the surface of each piece was polished with a sliding slicer. The surface morphologies of the glue line region of prepared samples were imaged by a scanning electron microscope (SU8010, Hitachi) under a high-vacuum operation mode at 5.0 kV (ref. ^26^). Before scanning electron microscopy, the vacuum-dried samples were pre-sprayed with gold–palladium (Au–Pd) by vacuum sputtering.

### Compositional analysis of biomass

The compositional analysis of biomass and substrates after lignin extraction followed the standard National Renewable Energy Laboratory method^27^. After two-step hydrolysis, the resultant solution was filtered and the filtrate was used for sugar analysis by ion chromatography (Thermo (Dionex), ICS-5000^+^). The precipitate was washed with water, dried at 105 °C and then weighed to determine Klason lignin content. Compositional analysis results are presented in Extended Data Table 2.

## Online content

Any methods, additional references, Nature Portfolio reporting summaries, source data, extended data, supplementary information, acknowledgements, peer review information; details of author contributions and competing interests; and statements of data and code availability are available at 10.1038/s41586-023-06507-5.

## Figures and Tables

**Extended Data Table 1:**
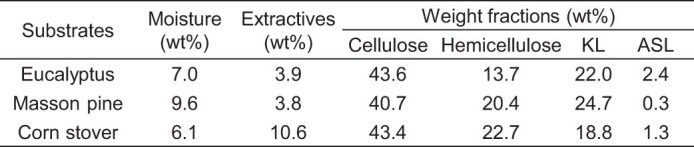
The analysis of variance (ANOVA) of factors affecting the bonding strength of multi-layer plywoods

ns: Not significant at *p* >  0.1; *: Significant at 0.05 ≤ *p* ≤ 0.1; **: Significantly different at *p* ≤ 0.05; ***: Significantly different at *p* ≤ 0.01.

**Extended Data Table 2:**
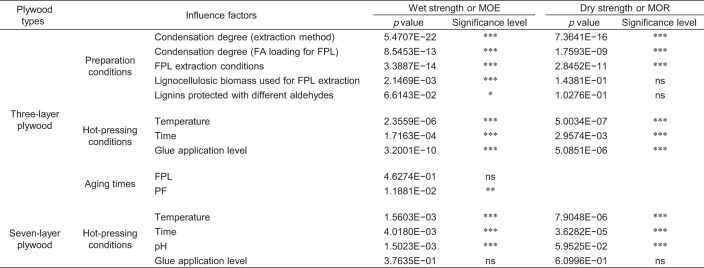
Compositional analysis of lignocellulosic biomass

The moisture contents (wt%) of lignocellulosic biomass were directly measured on the basis of air-dried biomass. The mass percentage contents (wt%) of extractives and other components were calculated on the basis of oven-dried extracted lignocellulosic biomass. “KL” refers to Klason lignin; and “ASL” refers to acsid-soluble lignin.

## Data Availability

The data supporting the findings of this study are available within the paper. Additional data are available from the corresponding author on reasonable request.
